# *MEF2C* Common Genetic Variation Is Associated With Different Aspects of Cognition in Non-Hispanic White and Caribbean Hispanic Non-demented Older Adults

**DOI:** 10.3389/fgene.2021.642327

**Published:** 2021-07-27

**Authors:** Preeti Sunderaraman, Stephanie Cosentino, Nicole Schupf, Jennifer Manly, Yian Gu, Sandra Barral

**Affiliations:** Cognitive Neuroscience Division of the Taub Institute for Research on Alzheimer’s Disease and the Aging Brain, G.H. Sergievsky Center, and the Department of Neurology, Columbia University Medical Center, New York, NY, United States

**Keywords:** memory, processing speed, language, visuospatial ability, cognition, genetic architecture, Alzheimer’s disease

## Abstract

**Objectives:**

Myocyte Enhancer Factor 2C (*MEF2C*) is identified as a candidate gene contributing to the risk of developing Alzheimer’s disease. However, little is known about whether *MEF2C* plays a role in specific aspects of cognition among older adults. The current study investigated the association of common variants in the *MEF2C* gene with four cognitive domains including memory, visuospatial functioning, processing speed and language among non-demented individuals.

**Method:**

Participants from two ethnic groups, Non-Hispanic White (NHW; *n* = 537) and Caribbean Hispanic (CH; *n* = 1,197) from the Washington Heights-Inwood Community Aging Project (*WHICAP)* study, were included. Genetic association analyses using WHICAP imputed genome-wide data (GWAS) were conducted for the various cognition domains.

**Results:**

Single nucleotide polymorphisms (SNP) variants in the *MEF2C* gene showed nominally significant associations in all cognitive domains but for different SNPs across both the ethnic groups. In NHW participants, the strongest associations were present for memory (rs302484), language (rs619584), processing speed (rs13159808), and visuospatial functioning (several SNPs). In CH, strongest associations were observed for memory (rs34822815), processing speed (rs304141), visuospatial functioning (rs10066711 and rs10038371), and language (rs304153).

**Discussion:**

MEF2C variant-cognitive associations shed light on an apparent role for *MEF2C* in both memory and non-memory aspects of cognition in individuals from NHW and CH ancestries. However, the little overlap in the specific SNP-cognition associations in CH versus NHW highlights the differences in genetic architectural variations among those from different ancestries that should be considered while studying the *MEF2C* gene.

## Introduction

The Myocyte Enhancer Factor 2C (*MEF2C*) gene located on chromosome 5 (5q14.3), belongs to a family of human transcription factors consisting of four proteins (that play central roles in the differentiation, morphogenesis, and maintenance of vertebrate tissues (Harrington et al., 2016; Pon and Marra, 2016). Studies in mouse models demonstrated that *MEF2C* is strongly expressed in several brain areas (hippocampus, dentate gyrus, amygdala, etc.) (Rashid et al., 2014) and it may modulate the reduction of dendritic spine growth (Rashid et al., 2014; Tang et al., 2016). In mice, the deletion of *MEF2C* gene was linked to impairments in learning and memory (Barbosa et al., 2008).

In humans, the *MEF2* family of transcription factors has been implicated in gene expression regulation critical for tissue integration, synaptic development and regulation of neuronal excitability (Assali et al., 2019). *MEF2C* expression has been reported in multiple brain regions including the hippocampus, basal ganglia, and amygdala (Uhlén et al., 2015). Genetic studies in children have found that *MEF2C* deletions are associated with several developmental disorders and intellectual impairments (Le Meur et al., 2010; Novara et al., 2013). Studies in older adults have linked variation in *MEF2C* gene to the risk of multiple neurological disorders, such as Late Onset Alzheimer’s disease (LOAD) and Parkinson’s disease (She et al., 2012; Dietrich, 2013; Davies et al., 2015).

Neuropathological studies have found *MEF2C* to be associated with Braak stage, capillary Aβ and neuritic plaques suggesting a contributory role of this gene to Alzheimer’s disease and Lewy body disease (Beecham et al., 2014; Mäkelä et al., 2018). In a large, meta-analysis of GWAS studies examining participants from European ancestry (45 years or older; *n* ∼53,949) without prevalent dementia, *MEF2C* was significantly associated with a cognitive function composite score consisting of tests from different domains including memory, processing speed, and executive functioning (Davies et al., 2015). However, despite *MEF2C*’s association with LOAD risk (Lambert et al., 2013), relatively little is known about the role of *MEF2C* in specific aspects of cognition such as memory, processing speed, language and visuospatial functioning in older adults.

Genetic variants associated with LOAD risk can potentially be used as predictive biomarkers for dementia if these variants are also associated with cognition in healthy older adults prior to onset of cognitive impairment (Pedraza et al., 2014). For example, the apolipoprotein E (APOE), highly associated with LOAD risk, has been found to be strongly associated with memory in healthy adults before the clinical manifestation of Alzheimer’s disease (Caselli et al., 2009). Little is known about the *MEF2C*’s associations with specific cognitive domains, and therefore, the current study aimed to investigate the association between *MEF2C* and cognitive performance in four cognitive domains including memory, visuospatial functioning, processing speed, and language. We separately conducted this analysis in two, non-demented groups consisting of those from a Non-Hispanic White (NHW) ancestry and those from a Caribbean Hispanic (CH) ancestry.

## Methods

### Participants

Non-demented adults aged 65 and older living in northern Manhattan were drawn from The Washington Heights-Hamilton Heights-Inwood Community Aging Project (*WHICAP*) (Tang et al., 2001). The current study used WHICAP participants with NHW and CH ancestries for whom cognitive testing was administered every 18 to 24 months with each session typically lasting for about 45–90 min (Tang et al., 2001). For obtaining detailed information about the WHICAP cohort, readers are further referred to Lipnicki et al. (2017), Lee et al. (2018), and Stern et al. (1992). All WHICAP participants provided written informed consent and the study procedures were approved by the Institutional Review Boards at Columbia University.

### Assessment of Cognitive Function

As previously reported in other studies, WHICAP participants were administered a standardized battery of tests to examine performance across 4 cognitive domains [see Stern et al., 1992; Siedlecki et al., 2009; Siedlecki et al., 2010 for methological details about how the cognitive factors were derived]. Briefly, to identify the underlying factor structure of the cognitive protocol, an exploratory factor analysis (EFA) using principal axis factoring and oblique rotation was performed on 15 variables of interest. The model derived from the EFA was converted to a simple structure Confirmatory Factor Analysis model, in which each variable loaded only on the factor that had the highest loading. The cognitive domains were quantified as composite scores of standardized measures of the individual neuropsychological tests. Raw scores were standardized using the sample’s means and standard deviations from the entire WHICAP sample at baseline. Standardized scores were then averaged to create specific domains. Supplementary Table 1 summarizes the individual test within each cognitive domain.

### Genome-Wide Association Genotype Data (GWAS)

WHICAP genome-wide genotyping was performed on the Illumina HumanHap 650Y and Omni 1M chips, according to Illumina procedures at Columbia University. Genotype calling was performed using GenomeStudio v.1.0. The physical positions of the reported SNPs are based on GRCh37 genome assembly. Quality control lead to removal of samples with call rates below 95%, sex discrepancies and relatedness. Genotype imputation was performed using IMPUTE2 with the 1000 Genomes Phase I NCBI Build b37 (June 2011) reference panel. SNPs were removed for analysis purposes if the imputation quality score was less than 0.8 or the minor allele frequency was less than 1%.

### Allele Frequencies and Linkage Disequilibrium

To investigate the allele frequencies and linkage disequilibrium patterns for *MEF2C* gene, we used LDpop Tool in LDlink (Machiela and Chanock, 2015).

LDlink is a suite of web-based applications to interrogate linkage disequilibrium across a variety of ancestral population groups. To calculate population-specific measures of linkage disequilibrium, LDlink accepts input for bi-allelic variants and uses publicly available reference haplotypes from Phase 3 (Version 5) of the 1000 Genomes Project. The current implementation of LDlink references The Single Nucleotide Polymorphism Database build 151 (dbSNP 151 released October 2017). LDlink modules report the following measures of linkage disequilibrium:

(i) D prime (D′ = 0–1), an indicator of allelic segregation for two genetic variants. D′ = 0 indicates no linkage of alleles; D′ = 1 indicates at least one expected haplotype combination is not observed.
(ii) R squared (*R*^2^ = 0–1), a measure of correlation of alleles for two genetic variants sensitive to allele frequency. *R*^2^ = 0 indicates alleles are independent, *R*^2^ = 1 indicates an allele of one variant perfectly predicts an allele of another variant.

The LDpop tool outputs an interactive color-coded map with the chosen linkage disequilibrium measure between SNP variants for the selected 1000G populations. LD plots were generated for the set of SNPs present in both NHW and CH ethnic groups. After excluding variants whose genomic positions did not match rs identifier at 1000G position or/and db151 search coordinates, the total number of SNPs for LD computation consisted of 297 SNPs.

The SNPs included in the analyses (Supplementary Figure 1) shown a strong pattern of LD, which appeared to be consistent across the different population groups considered.

### Statistical Analysis

Analyses were restricted to individuals with either NHW or CH ancestry having cognitive, demographic, and genetic data required for statistical analysis. Although the analyses may be impacted by the limited sample size and the compromised statistical power (Hong and Park, 2012), they are critical for establishing a potential domain specific role of MEF2C in determining cognitive functioning and for understanding whether specific associations vary by ethnic group.

Genetic associations with cognitive data were conducted using cognitive data from the last follow-up evaluation in order to examine the cognitive data most representative of the course of an individual’s cognitive aging, and most proximate to a potential change in cognitive status. The final set of single nucleotide polymorphisms (SNP) coverage of *MEF2C* gene, after quality-control metrics (excluding SNPs showing significant deviations from Hardy-Weinberg equilibrium and non-biallelic variants), consisted of 301 variants for NHW and 266 variants for CH. Based on the number of variants tested within each ethnic groups, *p*-values thresholds for experiment-wise significant were set as 1.7 × 10^–4^ for NHW and 1.9 × 10^–4^ for CH.

### Single SNP Association Analysis

Linear regression models were used to assess the association between genetic variants in the *MEF2C* gene and each cognitive domain. Linear models were adjusted for age, sex, education, and *APOE*_E4 status. For statistical analyses, *APOE* genotype was dichotomized into carriers (E4/E4, E4/E3) and non-carriers of the E4 allele (E2/E2, E2/E3 and E3/E3). All analyses were conducted using PLINK and IBM SPSS Statistics 25 software (Purcell et al., 2007).

## Results

Table 1 presents baseline demographic and clinical characteristics of the participants.

**TABLE 1:** Characteristics of study participants.

**Characteristics**	**Non-Hispanic white**	**Caribbean Hispanic**
	
	**Mean ± SD**	
Sex (n;% for women)	313 (60.3%)	836 (69.8%)
Age	83.62 ± 6.44	81.11 ± 6.45
Education	13.24 ± 3.5	7.22 ± 4.32
APOE_E4 carriers (%)	98 (18.9%)	258 (21.6%)

### Memory

In NHW participants, the strongest nominal association was observed for a cluster of SNP variants rs302484 and rs302482 (*B* = 0.33, *SE* = 0.17, *p* = 0.051). In CH participants, another SNP located 121Kb upstream of rs302484 yielded the strongest association (rs34822815, *B* = −0.31, *SE* = 0.12, *p* = 0.011). Table 2 shows the top nominal SNP-cognition associations for NHW and CH; only the top nominal SNP-cognition associations for NHW and CH are bolded in the table.

**TABLE 2:** Top nominal significant SNPs associations within each cognitive domain for Non-Hispanic White and Caribbean Hispanic participants.

			**NHW**			**CH**		
**Domain**	**SNP**	**bp**	***B***	***SE***	***p***	***B***	***SE***	***p***
Memory	rs302484	88,143,749	**0.33**	**0.17**	**0.051**	0.03	0.04	0.466
	rs302482	88,144,234	**0.33**	**0.17**	**0.051**	0.03	0.04	0.501
	rs34822815	88,022,379	0.33	0.21	0.120	**−0.31**	**0.12**	**0.011**
Language	rs619584	88,078,325	**0.09**	**0.04**	**0.027**	**−**0.03	0.02	0.178
	rs160044	88,114,476	0.03	0.04	0.443	**−0.08**	**0.02**	**3.5E-04**
	rs167345	88,118,858	0.03	0.04	0.493	**0.08**	**0.02**	**3.0E-04**
	rs304154	88,120,416	0.03	0.04	0.493	**0.08**	**0.02**	**3.5E-04**
	rs304153	88,121,188	0.03	0.04	0.493	**−0.09**	**0.02**	**2.6E-04**
	rs304152	88,124,123	0.02	0.04	0.571	**0.08**	**0.02**	**4.0E-04**
Speed	rs13159808	88,194,669	**−0.18**	**0.08**	**0.020**	**−**0.05	0.06	0.421
	rs304141	88,188,058	0.02	0.09	0.791	**0.21**	**0.07**	**0.002**
Visuospatial	rs2362108	88,147,771	**−0.06**	**0.03**	**0.041**	0.08	0.03	0.002
	rs2362109	88,148,041	**−0.06**	**0.03**	**0.041**	0.08	0.03	0.002
	rs2362110	88,148,638	**−0.06**	**0.03**	**0.041**	NA	NA	NA
	rs2362111	88,148,710	**−0.06**	**0.03**	**0.041**	0.08	0.03	0.002
	rs17487515	88,149,291	**−0.06**	**0.03**	**0.041**	0.08	0.03	0.002
	rs17559709	88,149,326	**−0.06**	**0.03**	**0.041**	0.08	0.03	0.002
	rs4145738	88,150,289	**−0.06**	**0.03**	**0.041**	0.08	0.03	0.002
	rs10066711	88,190,604	**−**0.04	0.03	0.149	**0.09**	**0.03**	**0.001**
	rs10038371	88,199,223	**−**0.04	0.03	0.141	**0.09**	**0.03**	**0.001**

*Bp, basepair; SE, standard error; SNP, single nucleotide polymorphisms.*

*Only the top nominal association with the lowest *p* values and highest *b* values have been bolded.*

### Processing Speed Domain

Among NHW, the strongest nominal associations was observed for SNP variant rs13159808 (*B* = −0.18, *SE* = 0.08, *p* = 0.020). Although the same variant was not associated with processing speed in CH, a SNP located 135 Kb upstream, yielded the strongest association with speed (rs304141, *B* = 0.21, *SE* = 0.07, *p* = 0.002).

### Visuospatial Domain

Among NHW, a cluster of several SNPs was nominally associated with worse performance (rs2362108 to rs4145738, *B* = −0.06, *SE* = 0.03, *p* = 0.041). In CH, the strongest association was observed for two other SNPs located downstream of rs2362108 (∼47 Kb) from rs10038371, *B* = 0.09, *SE* = 0.03, *p* = 0.001 for both SNPs, see Table 2 (Note: Only the top nominal SNP-cognition associations for NHW and CH are bolded in the table).

### Language Domain

Among NHW, a variant rs619584 was nominally associated with language (*B* = 0.09, *SE* = 0.04, *p* = 0.027). Among CH, a different SNP located 42 Kb upstream rs619584, yielded the strongest association at rs304153, *B* = −0.09, *SE* = 0.02, *p* = 2.6 × 10^–4^. None of reported associations reached the established thresholds for experiment-wise significance.

Supplementary Tables 2, 3 summarizes the association results for the 301 in NHW and 266 in CH for *MEF2C* SNPs across each of the four cognitive domains for all study participants.

## Discussion

To our knowledge, this is the first study to comprehensively examine the association between common genetic variants in the *MEF2C* gene and cognitive performance in four different domains, including memory, processing speed, visuospatial functioning and language. In order to understand the generalizability of these findings across ethnic background, we investigated these associations simultaneously in NH Whites and Caribbean Hispanic using similar cognitive domains. First, we found that there were several unique, nominal associations between *MEF2C’*s *SNP* variants and various cognitive domains. Second, for any given cognitive domain, there were ethnic specific associations of *MEF2C* variants and specific cognitive domain performance. Below, we address the specific findings with the qualification that after adjusting for multiple testing, the genetic associations were nominally significant.

With regard to specific domains, we found associations between *MEF2C* variants and visuospatial functioning in both CH and NHW, with seemingly weaker associations for the domain in both ethnicities. Regarding processing speed, the presence of several SNPs’ associations with this cognitive domain (see Supplementary Tables for all the nominally significant associations) suggests that *MEF2C* may contribute to motor speed in older adults, a finding which may be in line with previous studies showing that *MEF2C* is associated with abnormal motor patterns (Novara et al., 2013; Vreèar et al., 2017). Finally, we detected novel associations between MEF2C and language performance.

It is notable that of the cognitive domains under investigation, *MEFC2* appears to have the weakest association with memory. To better understand these associations with non-memory domains, it would be informative to examine MEF2C in the context of focal AD variants such as posterior cortical atrophy (PCA) and the logopenic variant of primary progressive aphasia (lvPPA), as well as non-AD dementias including Lewy Body disease and frontotemporal dementia. In fact, MEF2C has been reported to be association with clinico-pathological features of Lewy body disease (Beecham et al., 2014). Alternatively, studying MEF2c in relation to more precise elements of normal cognitive functioning in each domain (e.g., semantic knowledge versus fluency in the language domain, or perception versus construction in the spatial domain), would perhaps allow for a clearer understanding of the way in which MEF2C influences specific brain networks. Examination of MEF2C at both ends of the cognitive spectrum will provide interesting avenues for understanding its mechanistic role.

Previously, the rs700588 SNP has been found to be associated with neuropathological features in those with AD. It is interesting that we did not find a cognitive association with this SNP. Perhaps, this is because some SNPs that play a role in cognition may be different from other SNPs that play in the biological processes. Although this needs to be tested empirically, there is some evidence to suggest that various neuropathological features (cerebral amyloid angiopathy, Braak stage, etc.) are differentially associated with specific genetic loci (Beecham et al., 2014; Mäkelä et al., 2018).

Different SNPs were associated with cognition in NHW and CH. The purpose of the study was not to compare genetic associations between the ethnic groups, but rather to better characterize the cognitive phenotypes associated with MEF2C across two different ethnic groups. It is well-known that population-based studies are biased for not having demographically comparable cohorts, thus making the interpretation of the results complicated (Sirugo et al., 2019). The differential SNP associations across ethnic groups is expected based on the reported differences in the genetic architecture of human populations (Grinde et al., 2019; Sirugo et al., 2019). For example, a *MEF2C* SNP (rs190982) was associated with LOAD risk in Caucasians, but not in Han Chinese (Tang et al., 2016). The observation that the associated variants may have inconsistent direction of effect (positive effect in NHW and a negative effect in CH or vice versa) is not atypical and most likely attributable to population-specific linkage disequilibrium patterns. It is also possible that the differences in MEF2C-cognition association across ethnic groups may be due to socio-demographic differences between the NHW and CH. For example, the education level between the two groups varies considerably (Genovese et al., 2010).

Our study has some limitations. First, given the power concerns associated with a relatively small sample size, the primary analysis examined data at only the last evaluation. Longitudinal studies using a larger sample size with multiple follow-up visits and more comprehensive analytic methodologies (e.g., Latent Class models) are needed to examine whether the same genetic effects are reproducible when considering cognitive performance over time. Second, we have examined older adults with NHW and CH ancestries, therefore it will be necessary to generalize these findings in other racial/ethnic groups across other study cohorts. Third, among the cognitive domains, data for others such as executive function was not collected in the present study. Future studies will benefit by examining this construct’s association with *MEF2C* variants. Finally, rare genetic variants and potential interactions (epistatic and gene-environment) that might contribute to cognitive phenotypes variability were not considered in our analyses (Fan et al., 2019).

Despite the limitations, our study offers critical insights into the associations between MEF2C and various cognitive domains in healthy older adults and has implications for future research. It is known that *MEF2C* plays a role on inhibitory and excitatory neuronal activity, and as a transcription factor regulates other genes. The current study found *MEF2C* to be associated with different cognitive functions across two ethnic groups. Although our findings for the MEF2C-cognitive associations are nominally significant, they suggest that in a potentially larger, well-powered sample this gene may demonstrate stronger effects on cognition. It will also be interesting to examine whether *MEF2C* studied in the context of other genes provides a stronger biomarker signal for dementia prediction than when examined in isolation. Finally, given the increasingly important role of *MEF2C* in psychiatric disorders such as schizophrenia and depression, future studies can study the relation of this gene with specific types of dementias such as Lewy body dementia, behavioral variant frontotemporal dementia, and Alzheimer’s disease with comorbid psychosis.

## Data Availability Statement

The Washington Heights-Inwood Columbia Aging Project (WHICAP) welcomes manuscripts and papers based on project data and materials. Proposals are required for all papers and abstracts that use WHICAP data. This web survey includes fields for all the items needed for a proposal to be reviewed by the Publications Committee. Please complete and submit the form. Questions can be sent to NS, ns24@columbia.edu or JM, jjm71@columbia.edu. IRB approval is required for proposed analyses, and a Data Use Agreement or Material Transfer agreement may need to be obtained. A response must be entered in each field below. If a field is not applicable, please enter ‘N/A’. Please provide as much information as possible in response to each question. Text can be copied from other documents and pasted into the appropriate field. All text fields can be expanded as needed by dragging the hatched area in the bottom right corner of the field. WHICAP data and Material use policy. Acceptance of WHICAP data and/or materials obligates the recipient to cite/reference the NIA grants supporting this project (see required language below). Should publications result from the use of WHICAP data/materials now or in the future, the recipient agrees to notify the PI, Dr. Richard Mayeux, with details (reference or PubMed Central ID#) and provide a copy of the publication so that WHICAP may report productivity derived from our resources to the funding agency, the NIA. Such publications require compliance with National Institutes for Health (NIH) public access policies.

## Ethics Statement

The studies involving human participants were reviewed and approved by the Columbia University Medical Center. The patients/participants provided their written informed consent to participate in this study.

## Author Contributions

JM, NS, and SB contributed to data collection and compilation. PS, SC, YG, and SC contributed to data analysis. PS, SC, YG, NS, and SB contributed to the interpretation of the results and to the drafting of the manuscript. All authors participated to the approval of the final manuscript and took responsibility for the content and interpretation of this article.

## Author Disclaimer

The content is solely the responsibility of the authors and does not necessarily represent the official views of the NIH.

## Conflict of Interest

The authors declare that the research was conducted in the absence of any commercial or financial relationships that could be construed as a potential conflict of interest.

## Publisher’s Note

All claims expressed in this article are solely those of the authors and do not necessarily represent those of their affiliated organizations, or those of the publisher, the editors and the reviewers. Any product that may be evaluated in this article, or claim that may be made by its manufacturer, is not guaranteed or endorsed by the publisher.

## Supplementary Material

Supplementary Figure 1 Linkage Disequilibrium for the SNPs included in the analyses.

Supplementary Table 1 WHICAP individual cognitive tests under each cognitive domain derived from factor analyses.

Supplementary Table 2 Nominal asssociations of 301 SNPs in Non-Hispanic White participants across each of the four cognitive domains.

Supplementary Table 3 Nominal asssociations of 266 SNPs in Caribbean Hispanic participants across each of the four cognitive domains.
