# Supplementary figures and images for: MEF2C Common Genetic Variation Is Associated With Different Aspects of Cognition in Non-Hispanic White and Caribbean Hispanic Non-demented Older Adults
Source: Front Genet. 2021 Jul 27;12:642327. doi: 10.3389/fgene.2021.642327 (PMC8353395; doi:10.3389/fgene.2021.642327)

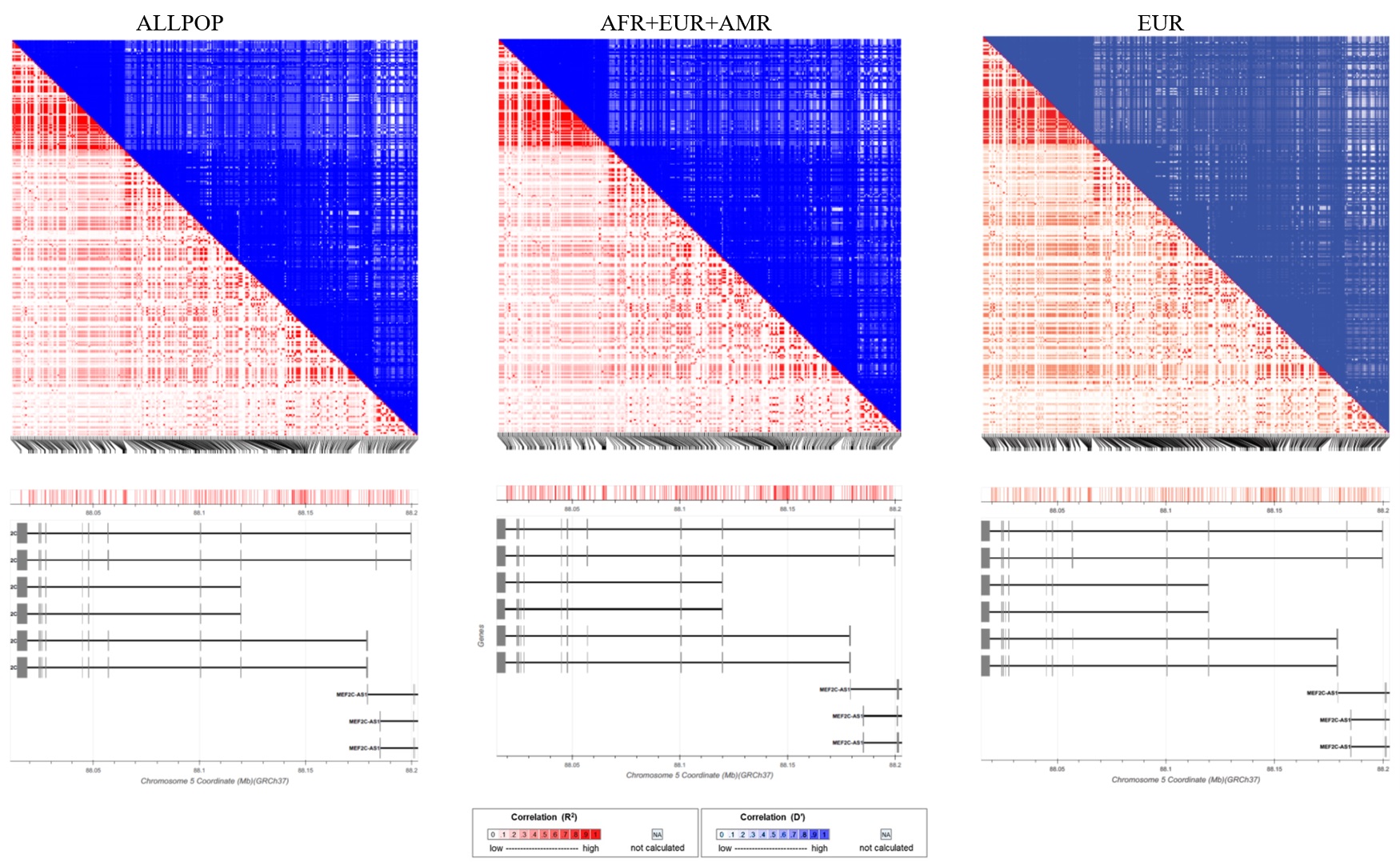

Supplement: Supplementary Figure 1 — Linkage Disequilibrium for the SNPs included in the analyses. [file Image_1.JPEG]
