# Supplementary material for: MEF2C Common Genetic Variation Is Associated With Different Aspects of Cognition in Non-Hispanic White and Caribbean Hispanic Non-demented Older Adults
Source: Front Genet. 2021 Jul 27;12:642327. doi: 10.3389/fgene.2021.642327 (PMC8353395; doi:10.3389/fgene.2021.642327)
Supplement: Supplementary Table 1 — WHICAP individual cognitive tests under each cognitive domain derived from factor analyses. [file Table_1.docx]

| **Supplementary Table 1. WHICAP individual cognitive tests under each cognitive domain derived from factor analyses.** | |
| --- | --- |
| Domain | Test |
| Memory | SRT-total recall  SRT-delayed recall  SRT-delayed recognition |
| Language | Naming based on modified 15-item BNT  Letter fluency (C, F, L)  Category fluency (Animals)  Similarities from WAIS-R  Repetition from BDAE  Comprehension from BDAE |
| Visuospatial | BVRT recognition  BVRT matching  Rosen Drawing Test (Rosen, 1981)  Identities/Oddities from DRS |
| Processing speed | Color Trails Test 1  Color Trails Test 2 |
| Note: BDAE = Boston Diagnostic Aphasia Evaluation (Goodglass, 1983); BNT = Boston Naming Test (Kaplan, Goodglass, & Weintraub, 1983); BVRT = Benton Visual Retention Test (Benton, 1955); DRS = Mattis Dementia Rating Scale (Mattis, 1976); SRT = Selective Reminding Test (Buschke & Fuld, 1974); WAIS-R = Wechsler Adult Intelligence Scale—Revised (Wechsler, 1981)  Note: Factor structures were derived in a previous study [21]. | |
